# Systemic Lupus Erythematosus with Multiple Autoimmune Disease Presented with Extensive Peripheral Gangrene

**DOI:** 10.1155/2020/8278275

**Published:** 2020-03-10

**Authors:** Tareq Z. Alzughayyar, Jihad Samer Zalloum, Mohammad N. Elqadi, Sadi A. Abukhalaf, Fawzy M. Abunejma, Mohammed M. Y. Alkhanafsa, Motasem H. M. Haif, Firas Alqarajeh, Mo'min R. Mesk, Rami A. Misk

**Affiliations:** ^1^Al-Quds University, Faculty of Medicine, Jerusalem, State of Palestine; ^2^Pediatric Rheumatologist, Al-Ahli Hospital, Hebron, State of Palestine; ^3^An-Najah National University, Faculty of Medicine & Health Sciences, Nablus, State of Palestine

## Abstract

Systemic lupus erythematosus (SLE) is an autoimmune disease and can be associated with other autoimmune diseases. SLE usually presents with skin change and rarely presents with gangrene. SLE gangrene usually involves the digits of upper extremities. We report the first case of SLE associated with an extremely rare constellation of neuromyelitis Optica (NMO) and diabetes mellitus type 1, presented with a rare form of the SLE gangrene which involves bilateral lower extremities up to midlegs, a case that has not yet been reported in the literature. Although SLE gangrene may respond to immunosuppressants, it has a high risk of complications that can end up with amputations.

## 1. Introduction

Systemic lupus erythematosus (SLE) is an autoimmune disorder leading to inflammation and tissue damage involving multiple organ systems and is more common in females with a 3 : 1 ratio [1]. SLE can present in a variety of clinical presentations. Skin manifestations are one of the most common presenting symptoms. However, gangrene is an exceptionally rare form of SLE cutaneous manifestations, accounting for 1.4% of SLE patients [2]. SLE gangrene most often affects the upper extremities and involves no more than the digits [3]. Pathophysiology underlying SLE gangrene includes vasospasm, thromboembolism, and vasculitis [4]. SLE vasculitis is uncommon and is usually small or medium-sized vessel vasculitis (9.7% and 1.6%, respectively), though large-vessel vasculitis are very unusual [5]. SLE is rarely associated with the autoimmune demyelinating Neuromyelitis Optica (NMO) which predominantly involves optic nerves and spinal cord. [6]. Herein, we report a 10-year-old female with multiple autoimmune diseases (SLE, NMO, and type 1 DM) presented with bilateral lower limb bluish cyanotic discoloration that rapidly developed into gangrene, necessitating bilateral below-knee amputations. This is the first case reported with such complex constellation in the literature as far as our knowledge and available data.

## 2. Case Presentation

**Figure 1:**
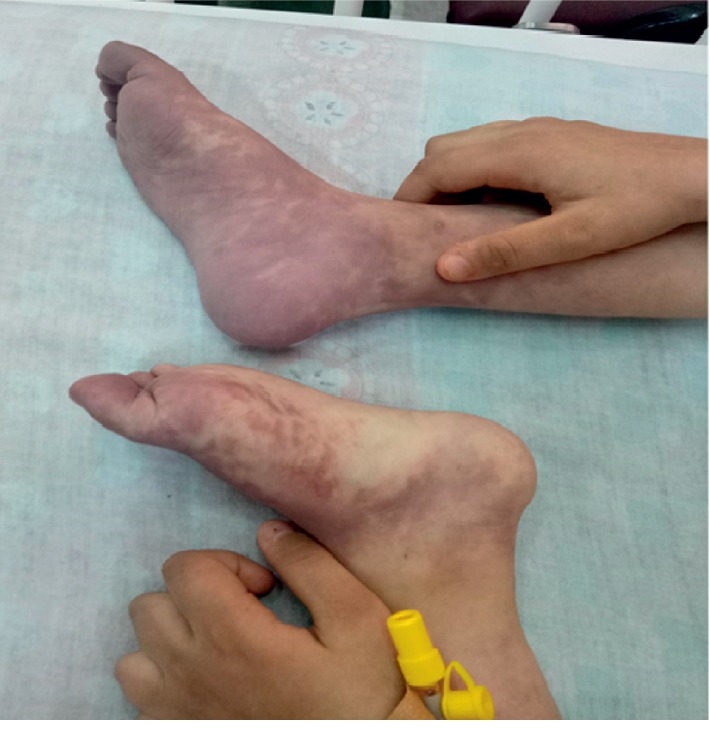
Bilateral bluish feet at admission.

A 10-year-old girl with a known history of systemic lupus erythematosus presented to the emergency department with acute painful bluish discoloration involving bilateral feet of rapid onset beginning 1 week ago (Figure 1).

Upon admission and during the physical examination, the patient had blue to red (violet) discoloration mainly involving feet, with the involvement of the hands and face, resembling maculopapular rash, and had active polyarthritis in the joints of the PIPs, MCPs, ankles, and right knee.

Lesions of the legs were bilateral below-ankle cyanotic changes with more severe and of wider spread in the right foot. No ulceration or gangrenous changes were noted. Dorsal pedis and posterior tibial pulses were sluggish and weak.

**Figure 2:**
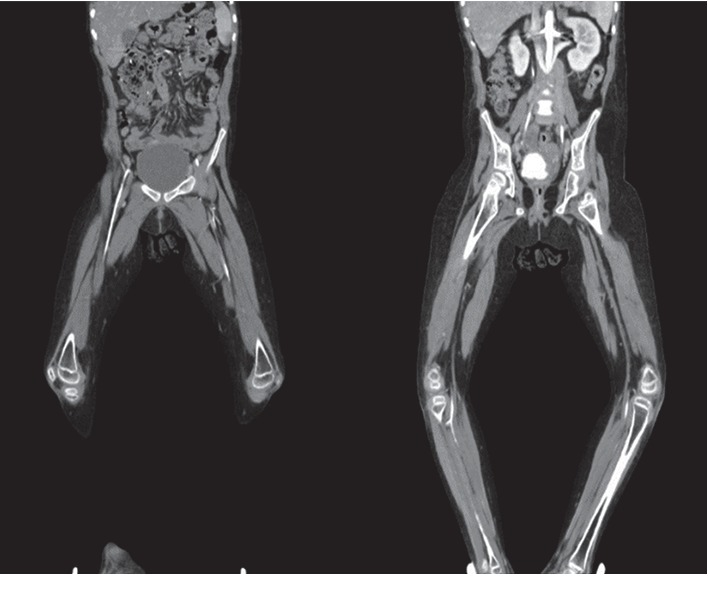
Contrast (white) shows weak blood flow in iliac, femoral, and popliteal arteries.

**Figure 3:**
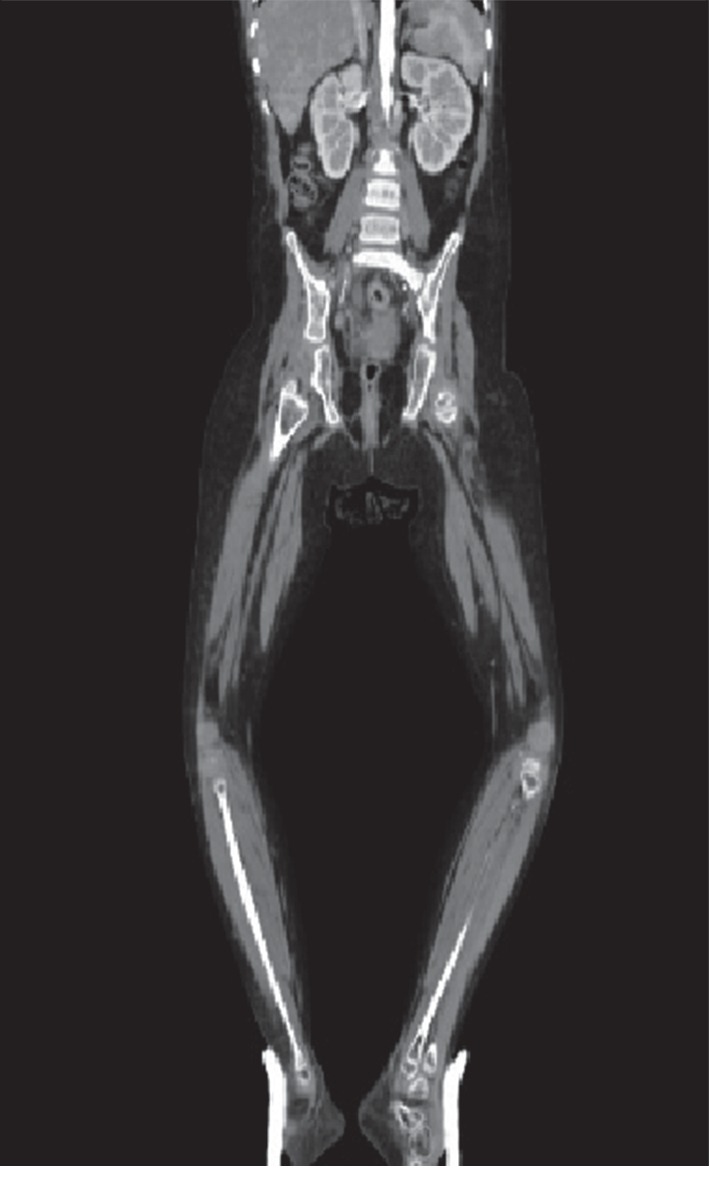
No contrast visible in distal popliteal and tibial arteries (no blood flow).

The patient was admitted, and Laboratory investigations were conducted (Tables 1 and 2). Blood and urine cultures were negative. Chest x-ray, echocardiography, foot x-ray, and abdominal sonography all were normal. Doppler ultrasound and CT angiography were positive (Figures 2 and 3).

IV Methylprednisone, IV Heparin infusion, IVIG, Methotrexate, and Antihypertensive drugs (carvedilol and Amlodipine) were given.

After treatment, proteinuria, c3, and c4 normalized, where ESR did not improve and remained around 80–110.

**Figure 4:**
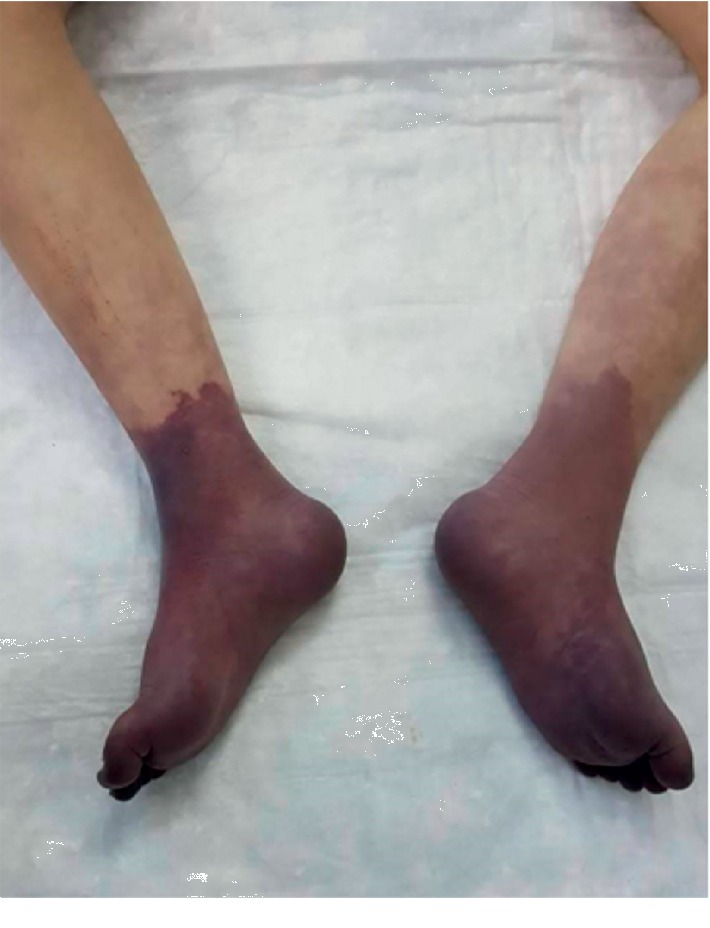
Gangrenous rash involves the whole feet and the lower part of the legs bilaterally.

During admission, pedal lesions progressed into dark purple discoloration and spread 4 cm above the ankle bilaterally (Figure 4). An increase in pedal pain and hypothermia were also reported. Examination showed absent pulse in distal popliteal, tibial arteries, and dorsal pedis arteries, which appears as ascending compromise of blood flow. Unfortunately, bilateral below-knee amputation warranted due to loss of viability.

In a previous visit to the Emergency department (days after onset, before admission), the patient reported the first symptom as sudden blue to red discoloration in the tip of the toes that rapidly spread to involve proximal portions of feet, associated with pain and hypothermia and has been treated as an outpatient with low-dose steroid, Azathioprine, MTX, Vitamin D, and Hydroxychloroquine.

Our patient was not only diagnosed with SLE at the age of nine according to SLICC (Systemic Lupus International Collaborating Clinics) and ACR criteria (Table 2) but also was known to have NMO from her first year of life and DM1 at the age of 7; the 10-year-old girl was paraplegic, blind, and in poor glycaemic control when presented to us, complicated by bilateral peripheral ischemia, which was confirmed by CT angiography (Figures 2 and 3) and was rapidly progressing to gangrene in spite of treatment.

## 3. Discussion

We report this case for its unusual constellation of different autoimmune pathologies. As far as we know and based on available data, there are no reports in the literature of SLE occurring in a child with NMO and type 1 DM, complicated by peripheral gangrene.

SLE is an autoimmune multisystemic disease, linked to considerable morbidity and mortality but no known etiology [7]. Cutaneous manifestations are valuable in regards to diagnosis and prognosis due to the fact of 7 out of 11 ARA criteria for SLE are cutaneous [8]. Digital ischemia, digital ulcer, and gangrenous lesions, though rare, it is previously reported in SLE.

Peripheral gangrene in SLE is very rare, especially if it involves large and medium-sized arteries. Occurring in about 1% of SLE patients, and most often affects the upper extremities [9–11], it is considered a severe complication that generally leads to amputation [12].

Risks factors for SLE peripheral gangrene include: long disease duration (≥4 years), Raynaud's phenomenon, and elevated serum C-reactive protein (CRP) [11]. Moreover, Anti-RNP may aid the development of digital gangrene as found to have an association with Raynaud phenomenon and APS [8, 13, 14].

Causes of SLE peripheral gangrene are unknown and multifactorial, and the underlying mechanism is complex and diverse, though can be summarized as follows: (1) vasculitis and infectious disorders that could cause vasculitis, (2) rheumatological disorders, (3) mechanical and obstructive disorder, (4) premature atherosclerosis, (5) vasospasm, (6) overlap syndrome, and (7) hypercoagulability-thrombosis related to antiphospholipid (APL) antibodies or embolus originating from the heart secondary to Libman-sack endocarditis, all of which may contribute to the development of gangrene [12, 15–22].

Inherited classical complement deficiencies can be associated with SLE as it shares some common immunological and clinical features, especially early age onset of cutaneous involvement. However, as reported in the literature, the presence of positive anti-dsDNA antibodies, negative anti-cardiolipin antibodies, and normalization of complement level after medical treatment does not support complement components deficiency [23, 24].

DM is also a possible cause of peripheral gangrene; though our patient had DM for 2 and half years, it is unlikely to be the causative agent in our case because of bilateral symmetrical involvement of both lower limbs and the very acute onset and deterioration within days.

Of the mentioned causes, vasculitis and APS are repeatedly linked with gangrene in the setting of SLE. Most of the reported cases indicate the presence of antiphospholipid antibodies, indicating that APS could be the largest contributor.

APS is significantly associated with gangrene in SLE patients (3.3–7.5%) [13, 25]. Increased ANA and Anti-*β*2 glycoprotein I autoantibodies were noted in some cases of peripheral gangrene [13, 26, 27].

Vasculitis in SLE occurs almost exclusively in small vessels. The primary pathology is leukocytoclastic vasculitis. Medium- and large-vessel vasculitis in association with SLE is unusual [16].

Recognizing the underlying cause of peripheral gangrene in SLE is aided by histopathology, imaging modalities, and serological markers.

Histopathology can reveille the underlying cause, vasculitis vs. thrombosis, or mixture of both (secondary vasculitis in APS) [9, 28]. Vasculitis signs can be demonstrated in the less-invasive modality CT angiography, namely, wall thickening, irregularity, and luminal narrowing. TEE detects vegetations of Libman sac endocarditis and can also aid diagnosis [29]. The serological marker can put light on the possibility of APS when antiphospholipid (APL) antibody is present.

In a nutshell, the diagnosis of underlying pathology is much of importance and can guide us into more clear management, vasculitis vs. hypercoagulability treatment.

Our patient had negative antiphospholipid (lupus anticoagulants, Anticardiolipin, and anti B2-glycoprotein) antibodies twice 6 months apart and no previous hx of any thrombotic event, CVA, or peripheral vascular events, and imaging wise there was no evidence of thrombosis; thrombophilia profile (protein C, S and Leiden v) was normal when she was investigated due to NMO and paraplegia, which makes thrombosis less likely to be the cause of this gangrene.

Treatment of SLE gangrene is generally divided into two classes:

1. Anticoagulation: heparin and warfarin for APS/LAC.
2. Immunosuppression: corticosteroid and immunosuppressive drugs for vasculitis [30]. Rituximab, low-dose epoprostenol, mycophenolate, and cyclophosphamide can also be used [7, 8, 31].

Liu et al. study suggested that early treatment with an aggressive corticosteroid, immunosuppressive drugs, lipid lowering agents, and anticoagulation therapies in patients complicated with antiphospholipid syndrome may prevent gangrene from deterioration, decrease the hazard of amputation, and improve prognosis in patients with SLE [11, 32].

Surgical resection is reserved for severe cases as our patient, who despite the combination therapy, deteriorated rapidly. However, many patients resolve without any complications, while some have auto amputations [9].

Concerning prognosis, some medical literature observed an unusual phenomenon that after gangrene, many patients entered a state of clinical remission and for unknown reasons could be a good prognostic sign in SLE. Or due to the fact that gangrene is such an alarming sign, early diagnosis and aggressive treatment could recover the balance of the immune system, avoiding future relapses [9, 10]. Lacking data on cases that progressed to death or severely deteriorated could falsely present this fact to us, and though controversial, it was worth mentioning [9].

Prevention included control of traditional and disease-related risk factors [33]. Also, patients of SLE vasculitis should avoid cold exposure due to the risk of gangrene [34].

In conclusion, despite their young age, SLE patients are at increased risk of developing peripheral ischemic gangrene, and diagnosis of underlying pathology is much of importance and can guide us into more clear management. Early and aggressive corticosteroid treatment prevented gangrene from progression and improved prognosis.

## Figures and Tables

**Table 1:** Routine laboratory tests.

Lap test	Value
ECR	100 mm/h
CRP	8 mg/l
HbA1c	9.6%
Random blood sugar	200 mg/dl
BUN	16 mg/dl
Creatinine	1 mg/dl
Aspartate aminotransferase (AST)	22 U/L
Alanine aminotransferase (ALT)	18 U/L

**Table 2:** New ACR and EULAR criteria scoring for our case.

Domain	Criteria	Points	Patient value	Patient score
ANA = 1 : 120				

*EULAR/ACR clinical domains and criteria for SLE*				
Constitutional	Fever	2	Positive	**2**
Hematologic	Leukopenia Thrombocytopenia Autoimmune hemolysis	3 4 4	WBC: 10,000 PLT: 233,000 Hb: 10 g/dl	0 0 0
Neuropsychiatric	Delirium Psychosis Seizure	2 3 5	Negative	0 0 0
Mucocutaneous	Nonscarring alopecia Oral ulcers Subacute cutaneous or discoid lupus Acute cutaneous lupus	2 2 4 6	Positive only for acute cutaneous lupus	0 0 0 **6**
Serosal	Pleural or pericardial effusion Acute pericarditis	5 6	Negative	0 0
Musculoskeletal	Joint involvement	6	Positive	**6**
Renal	Proteinuria >0.5 g/24 h Renal biopsy class II or V lupus nephritis Renal biopsy class III or IV lupus nephritis	4 8 10	0.51 g/24 hr negative negative	**4** 0 0

*EULAR/ACR immunologic domains and criteria for SLE*				
Anti-phospholipid antibodies	Anti-cardiolipin antibodies or Anti-*β*2GP1 antibodies or Lupus anticoagulant	2	Anti-cardiolipin IgM: 0.4 *μ*/ml Anti-cardiolipin IgG: 0.6 *μ*/ml Anti-*β*2GP1 IgM: 2.2 *μ*/ml Anti-*β*2GP1 IgG: 0.3 *μ*/ml. Lupus anticoagulants PTT: 30 seconds	0
Complement proteins	Low C3 or low C4 Low C3 and low C4	3 4	C3 = 33 C4 = 5	**4**
SLE-specific antibodies	Anti-dsDNA antibody or Anti-Smith antibody	6	Positive Anti-dsDNA: IgM = 200 IgG = 70	**6**

**Total patient score** **=** **28**				
